# An insight into recent developments of copper, silver and gold carbon dots: cancer diagnostics and treatment

**DOI:** 10.3389/fbioe.2023.1292641

**Published:** 2023-12-14

**Authors:** Ihsan Ullah, Hazrat Suliman, Muhammad Alamzeb, Obaid-Ur-Rahman Abid, Muhammad Sohail, Mohib Ullah, Abdul Haleem, Muhammad Omer

**Affiliations:** ^1^ Institute of Chemical Sciences, University of Swat, Swat, Pakistan; ^2^ Department of Chemistry, University of Kotli, Kotli, Pakistan; ^3^ Department of Chemistry, Hazara University, Mansehra, Pakistan; ^4^ Department of Chemistry, Balochistan University of Information Technology Engineering and Management Sciences (BUITEMS), Takatu Campus, Quetta, Pakistan; ^5^ School of Chemistry and Chemical Engineering, Jiangsu University, Zhenjiang, China

**Keywords:** cancer, nanotheranostics, bioimaging, phototherapy, doped carbon dots

## Abstract

Cancer is one of the most fatal diseases globally, however, advancement in the field of nanoscience specifically novel nanomaterials with nano-targeting of cancer cell lines has revolutionized cancer diagnosis and therapy and has thus attracted the attention of researchers of related fields. Carbon Dots (CDs)–C-based nanomaterials–have emerged as highly favorable candidates for simultaneous bioimaging and therapy during cancer nano-theranostics due to their exclusive innate FL and theranostic characteristics exhibited in different preclinical results. Recently, different transition metal-doped CDs have enhanced the effectiveness of CDs manifold in biomedical applications with minimum toxicity. The use of group-11 (Cu, Ag and Au) with CDs in this direction have recently gained the attention of researchers because of their encouraging results. This review summarizes the current developments of group-11 (Cu, Ag and Au) CDs for early diagnosis and therapy of cancer including their nanocomposites, nanohybrids and heterostructures etc. All The manuscript highlights imaging applications (FL, photoacoustic, MRI etc.) and therapeutic applications (phototherapy, photodynamic, multimodal etc.) of Cu-, Ag- and Au-doped CDs reported as nanotheranostic agents for cancer treatment. Sources of CDs and metals alogwith applications to give a comparative analysis have been given in the tabulated form at the end of manuscript. Further, future prospects and challenges have also been discussed.

## 1 Introduction

**Cancer** is the second biggest cause of human deaths after cardiovascular diseases (Bray et al., 2018). Even though significant advancement in cancer treatment has been achieved recently, however, aggressive breast-, lung- and pancreatic tumors with low survival rate suggests many efforts to be undertaken (Leonardi et al., 2018). Millions of people are diagnosed with cancer every year most oftenly in the last stages of this disease. Therefore, for effective cancer treatment timely and correct diagnosis is of crucial importance. Majority of anticancer drugs that are generally recommended for tumor enucleation induce terrible damage not only to the cancer cells, but also to other normal cells (Sunbal et al., 2023). One of the most important areas of cancer research is to improve and enhance the targeting efficacy of anticancer drugs over cancer cell lines. Recently, in this direction novel nanomaterials have aided in targeting cancer cells with overall low dosage, higher efficacy, minimal side effects and improved patients’ life quality (Khan et al., 2022; Ullah et al., 2022).

Nowadays, nanotechnology has emerged as a key player in cancer treatment and therefore different nanomaterials are studied for successful and improved imaging, sensing, drug delivery, therapy of cancer cell lines (Nocito et al., 2021; Ali et al., 2022). Like other nanomaterials, C-based quantum NPs have also the ability to refurbish cell fate, prevent or induce mutations, trigger cell–cell communication and revamp cell structure in a fashion required mainly during the phenomena at the bio-nano interface (Naik et al., 2022).

Since their accidental discovery in 2004, CDs have found huge biomedical applications due to their wide choice of precursors, low cost, facile synthesis, exceptional biocompatibility, fairly high shelf life, easy and tunable surface passivation, exclusive physical and optical properties, low or either nontoxicity, up-conversion photoluminescence and excitation wavelength-dependent FL emission in comparison to other carbon nanomaterials (Tejwan et al., 2021). Further, their enhanced photostability and brilliant PL intensity gives them superiority over other available semiconductor quantum dots (Hadad et al., 2021).

CDs can be easily and immediately excreted from the body through urine. With a neutral surface charge and hydrodynamic diameter of less than 5.5 nm carbon dots can efficiently pass through glomerular filtration without any harmful aggregation in spleen or liver (Nie, 2010; Pal et al., 2020). Due to these unique and state-of-the-art features, they are potential candidates in cancer treatment and have been successfully used in bioimaging, as a nano-drug carrier, drug and gene delivery and photothermal and photodynamic therapy etc (Kaurav et al., 2023). It has also been noted that all these properties of CDs are mainly dependent on the sources used, synthesis routes and it has been proved that these properties can be enhanced by surface modification and doping (Fang et al., 2017).

Recently, the development of heteroatom-doped Carbon Dots has gained enormous attention. Several non-metallic heteroatoms (N, S, P, B, F, etc.) as well as metallic heteroatoms (Ag, Au, Cu, Co, Fe, Ga, Gd, Mg, Mo, Ni, Sn, Zn, etc.) as dopants to increase the physicochemical properties of CDs. Some of these metals are essential elements for human body like Fe, Cu and Zn etc. while others like Au, Ag, Ga, La, etc. are eco-friendly and less toxic in nature making them potential dopants for CDs owing to their biomedical usefulness (Khera et al., 2010; Ullah et al., 2011a; Nawaz et al., 2011; Nawaz et al., 2012; Tejwan et al., 2021). These metal-doped carbon dots excrete very rapidly within 10 min post-injection through urine with complete elimination from body within 24 h in mice whether either administered through intramuscular, intravenous and subcutaneous injection routes (Huang et al., 2013).

As metals have larger atomic radius, better electron donation and higher number of unoccupied orbitals than non-metal heteroatoms therefore, metal ions doping could alter the electronic structure of CDs. This changes the HOMO-LUMO energy gap which shifts the FL from blue to red emission that determines the improved photo-optical and physicochemical properties of metal-functionalized doped carbon dots as compared with non-metallic heteroatom doping (Ullah et al., 2009; Ahmad Khera et al., 2010; Ullah et al., 2010a; Ibad et al., 2010; Ullah et al., 2011b; Ali et al., 2013; Hassan et al., 2013; Yue et al., 2023). In comparison to pristine carbon-dots the metal-functionalized CDs show intense optical absorbance in the visible region due to charge-transfer absorbance while enhanced FL is observed due to surface plasmonic resonance (SPR) of metal NPs (Sun et al., 2019).

Metal-doping of CDs generates new emission energy traps that brings about structural changes in carbon dots and better electron-hole purging which results in excellent quantum yield of CDs. As a result, multicolor emission under single excitation wavelength of 365 nm such as deep ultraviolet, blue, blue-green, green, yellow etc. is obtained (Zhai et al., 2018; Arshad and Sk, 2020). Metal doping also enhances the possibility for effective binding with redox species due to better charge distribution and spin density (Kar et al., 2023).

The biological and medicinal importance of group-11 metals (Copper (Safaei and Howell, 2005; Farrer and Sadler, 2008; Ali et al., 2009; Ullah et al., 2010b; Scheiber et al., 2013), Silver (George et al., 1997; Chopra, 2007; Soumya and Hela, 2013) and Gold (El-Sayed et al., 2005; Rosi et al., 2006; Dhar et al., 2009; Raubenheimer and Schmidbaur, 2014)) is well recognized. Apart from their medicinal importance these metals have high electrical conductivity and better electron-donating/accepting potential which makes them useful dopants resulting in comparatively enhanced applications of CDs (Jiao et al., 2022). Although some reviews have reported physical, chemical and biomedical applications of metal/non-metal-doped CDs. However, no such review is available on biomedical applications of Cu-, Ag- and Au-doped/hybrid CDs especially on cancer imaging, diagnosis and therapy. This review highlights imaging applications (FL, PA, MRI etc.) and therapeutic applications (phototherapy, photodynamic, multimodal etc.) of Cu-, Ag- and Au-doped CDs reported as nanotheranostic agents for cancer treatment. Further, future prospects and challenges have also been discussed at the end of manuscript.

## 2 Biomedical applications

The most encouraging applications of CDs have been reported in biomedicine. With no phenomenal signs of inflammation in rats (Nekoueian et al., 2019), the applications of CDs for cancer treatment has gained momentum in recent days.

**FIGURE 1:**
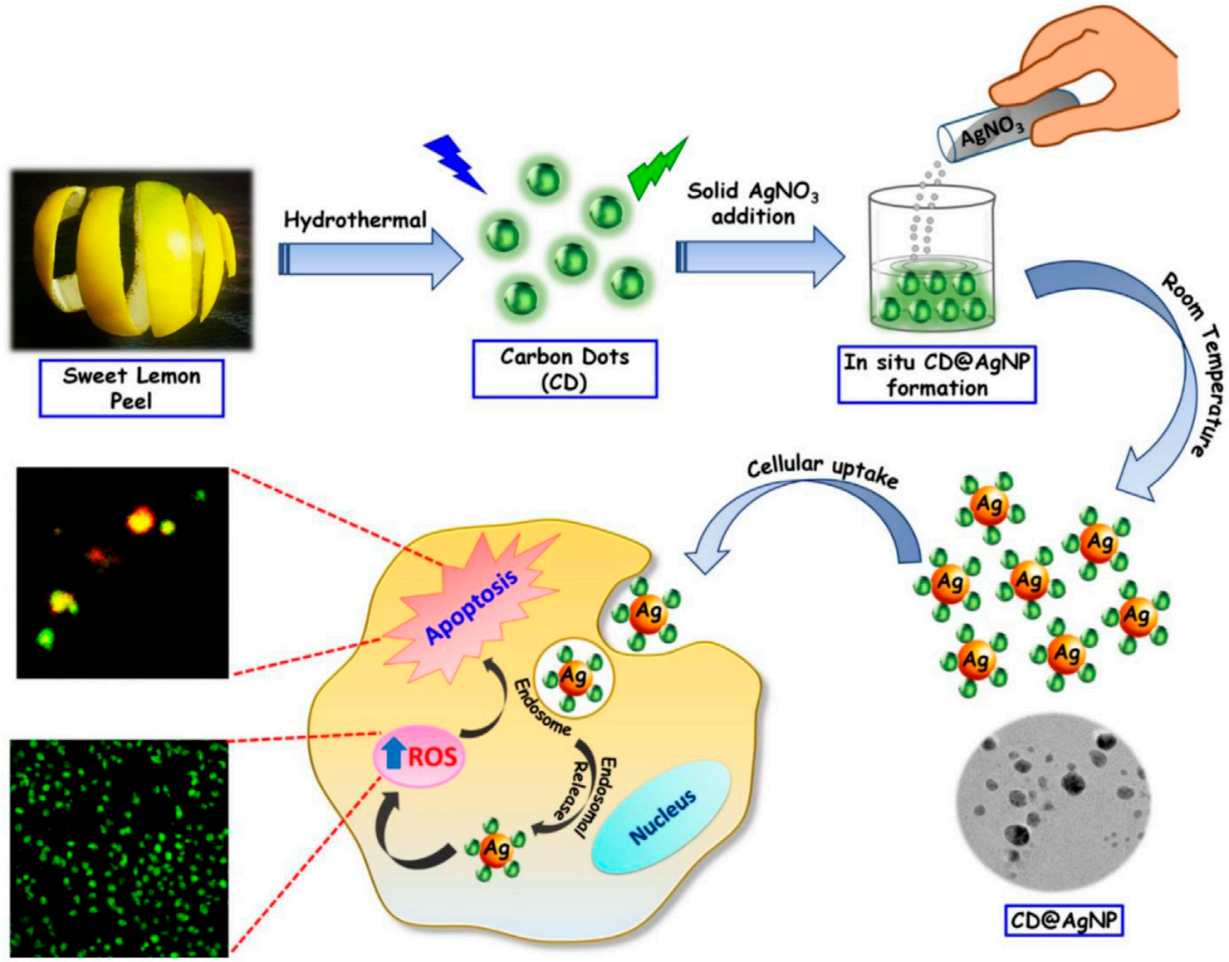
Representative Schematic presentation of group-11-CDs nanocomposites indicating stages of synthesis and applications (Ghosal et al., 2020): CD@AgNPs synthesis with their probable cellular activity toward breast cancer cells.

Copper (Cu), Silver (Ag), and Gold (Au) doped Carbon Dots (CDs) nanoparticles and nanocomposites can be synthesized employing a diverse range of preparation techniques, including hydrothermal (as shown in Figure 1), solvothermal, microwave, ultrasonic, laser ablation, carbonization, and pyrolysis. The precursor selection involves the utilization of suitable carbon source such as glucose, plant leaves and fruits, and various acids for CDs synthesis. Various sources of metals can be utilized such as CuCl_2_, CuSO_4_, and CuNO_3_ for Copper doping, while silver doping mainly utilizes AgNO_3_ as shown in Figure 1 and gold doping utilizes primarily HAuCl_4_, respectively.

These nanoparticles play a crucial role in multimodal imaging-guided photothermal cancer therapy. Both *in vivo* and *in vitro* studies demonstrate synergistic cancer therapies encompassing photothermal therapy (PTT), photodynamic therapy (PDT), chemotherapy (CT), chemodynamic therapy (CDT), among others. Additionally, they induce apoptosis (cell thermal death) by generating reactive oxygen species (ROS) or singlet oxygen (^1^O_2_) within cancerous cells as shown in Figure 1. Furthermore, the nanoparticles exhibit fluorescence imaging capabilities and facilitate cancer cell detection.

### 2.1 Bioimaging

Bioimaging is an established and facile technique that can give clear real-time and an unambiguous picture of biological taking use of different types of detectors and probes. There are various bioimaging techniques like B-ultrasound, Computed tomography (CT), Magnetic resonance imaging (MRI), Positron emission computed tomography (PET), X-ray and so on (Li et al., 2021). Among them FI, plays an important role for clinical diagnosis and bioimaging because of its cost-effective simple and convenient instrumentation, easy and long-term observation, high sensitivity and noninvasiveness. Until now, different fluorescent materials have been employed for bioimaging including small organic molecules and nanomaterials. However, typical fluorophores like organic dyes and quantum dots have the disadvantage of low FL performance and toxicity. Therefore, carbon dots have emerged as promising candidates as a replacement for conventional fluorescent probes both for biological *in-vitro* and *in-vivo* imaging with advantages as mentioned earlier (Li et al., 2017). Customarily, CDs can enter quickly into cells and are thus distributed into imaging organelles to study and understand organelle related diseases (Li et al., 2017). In addition, when conjugated with protoporphyrin IX, a nucleus photodynamic therapy capability was obtained to achieve efficient tumor removal after laser irradiation without any toxicity. Taking advantage of the minimal auto-FL and light straggling by tissues, CDs with red/NIR emission or two-/multiphoton photoluminescence have given best results for *in-vivo* FI (Yang et al., 2009). In addition, CDs have been identified to show outstanding multimodal imaging performance with an advantage to increase the efficiency of imaging-guided theranostics by visual information and guidance like circulation in physiological environments and location of agent (Liu et al., 2020; He et al., 2022).

In the following section Cu-, Ag-, and Au-doped CDs are discussed that have been used to achieve *in-vitro* and *in-vivo* bioimaging in the recent past.

#### 2.1.1 Cancer detection and imaging based on Cu-CDs

Bao et al. (2018) have developed CuCDs interconnected nanosheets that exhibit remarkable optical absorption in the NIR region (Entry-1 of Table 1). When modified with PEG, the resulting PEG-CuCDs NSs, which have a size range of 20–30 nm, exhibit good photothermal stability and biocompatibility. These nanosheets were utilized for a combination of imaging techniques and photothermal therapy to target and treat cancer cells effectively, Consisting of *in vivo* PA, Photothermal imaging also FL imaging *in vitro*. The high NIR optical absorption of the modified CuCDs NSs was used to investigate their PA properties, a direct correlation was noticed between the intensity of the PA signal and the concentration of copper, making them suitable for quantitative imaging using photoacoustic technique. Furthermore, after FL labeling and modification with HS-PEG, the CuCDs NSs were effectively reached tumor sites and target it by EPR phenomenon, where FI was used to guide photothermal therapy (PTT). Guo et al. (2018) used copper N-carbon dots both *in vivo* and *in vitro* as a FI probe and thermal imaging probe to render the therapeutic treatment process (Entry-2 of Table 1). The B16 cells were stained with Cu, N-CDs for cellular visualization to determine the Cu, N-CDs presence, within the cells. A bright FL was observed in the cytoplasm of the cell, confirming the entrance of Cu, N-CDs inside cell. Moreover, the fluctuations in tumor temperatures during laser exposure of 808 nm were recorded using an IR thermal camera. This shows that Cu, N-CDs can also be used as type of Infrared thermal imaging probe for monitoring the variations in temperature during PA process. Liu et al. (2019) developed a novel carbon nanostructure known as CBQDs using spinach, which exhibits near-infrared emission (Entry-3 of Table 1). Further binding of Cu ions leads to CBQD-Cu NCs. *In vivo* this NC demonstrated a capability for NIR FI of Biothiol. Since in cancerous cells the level of Biothiol is significantly higher as compare to normal cells, CBQD-Cu NCs have the potential to differentiate between cancerous and normal cells, enabling cancer diagnosis. The study used 7702 and HeLa cells for FI, where Biothiol emitted red FL after incubation with CBQD-Cu NCs and with a laser irradiation of 405 nm. They also validate the lower intensity of red FL exhibited by 7702 cells compared to HeLa cells. These findings indicate the potential of CBQD-Cu NCs for cancer diagnosis. Wang et al. (2019) describes synthesis and characterization of Cu-CDs that are suitable for FI in case of both HeLa and human neuroblastoma cells line (Entry-4 of Table 1). Confocal laser scanning microscopy (CLSM) was employed to capture FI of Cu-CDs in both SH-SY5Y multicellular spheroids (MCs) and HeLa cells. Which showed a 24.4% FL quantum yield. The Cu-CDs displayed favorable solubility, intense FL, and minimal cytotoxicity making them a promising candidate for optical bioimaging applications Ming Zhang, Wentao Wanga et al. synthesized a biodegradable, versatile NP system called γ-PGA @GOx @Mn, Cu-CDs for synergistic cancer therapy and simultaneous multimodal imaging, including FI, MRI, photoacoustic (PA) and ultrasound imaging. Briefly the study showed that the 4T1 tumor had a higher FL intensity compared to other organs. This indicates that the NPs exhibited a preference for accumulation in the tumor tissue. The signal strength within the tumor area on MRI images was also observed to be heightened after the injection of NPs, the photoacoustic (PA) images provided further evidence of NP accumulation within the tumor tissue with maximum accumulation observed after 36 h. Finally, the ultrasound imaging shows the production of oxygen (O_2_) within the tumor. The experiment revealed a progressive increase in oxygen (O_2_) levels within the tumor following NP injection (Liu et al., 2020) (Entry-5 of Table 1). Jiang et al. (2020) successfully synthesized a versatile nano platform known as CuO@CNSs-DOX, which combines CuO and CNSs for dual-modal imaging techniques namely, Infrared thermal imaging and photoacoustic imaging to provide the live diagnosis of a diseases (Entry-6 of Table 1). The IR thermal imaging results demonstrated that the application of CuO@CNSs and exposure to an 808-nm laser led to a 20°C rise in the tumor surface temperature. In contrast, the control group exhibited a temperature increase of 10°C. This result suggests that CuO@CNSs can effectively induce hyperthermia in tumors. The CuO@CNSs have been demonstrated to be a highly effective as a contrast agent in facilitating image-guided cancer diagnosis, particularly in photoacoustic (PA) imaging. *In vitro* studies showed a strong linear relationship between the concentration of CuO@CNSs and PA signal, CuO@CNSs have shown promising potential as a highly effective contrast agent for PA imaging. *In vivo* PA imaging also confirmed the effectiveness of CuO@CNSs as a contrast agent, Prior to injection, the tumor region exhibited weak PA signals, but after intratumoral administration of CuO@CNSs, strong PA signals were observed in the same area. These results suggest that CuO@CNSs can be an important tool for real-time imaging-guided cancer diagnosis. Sun et al. (2020) developed a nanoassembly called Cu/CC NPs by combining a photosensitizer called (chlorine e6 (Ce6), modified CDs (CDs-Ce6) and Cu^2+^ (Entry-7 of Table 1). These NPs were found to have exceptional capabilities for tumor microenvironment (TME) triggered FI. Confocal laser scanning microscopy (CLSM) was employed *in vitro* to investigate the FI of Cu/CC NPs in relation to three distinct carcinoma cell lines. The results showed that the Cu/CC NPs efficiently penetrated the cells and had a TME-stimuli responsive FL recovery, as evidenced by the bright red emission of the 4T1 cells that could be easily distinguished in the CLSM images. Additionally, nucleus-targeted staining was performed using Hoechst dye, demonstrating the suitability of the nanoassemblies for effective counterstaining. Comparable imaging properties were likewise observed for A549 and MCF-7 cells. The effect of GSH on FI using Cu/CC NPs was also investigated. When 4T1 cancerous cells were pre-cultured with acids known as alpha lipoic acid, which act as promoter for GSH, there was a 1.8-fold increase in FL intensity compared to untreated cells. This result was consistent with FL recovery seen after the GSH introduction. This indicates that in living cells the GSH can enhance the FI performance of the nano-assemblies. *In vivo* imaging the study investigated the effects of Cu/CC NPs, which showed excellent TME-triggered FI at the cellular level. Hemolysis experiments were performed to examine the blood compatibility of the NPs, which showed no significant damage to red blood cells, indicating their safety for use *in vivo*. The biodistribution of Cu/CC NPs was evaluated by employing an IVIS spectrum imaging system, which demonstrated a remarkable propensity of the nanoassemblies to accumulate efficiently at tumor site. Giang et al. (2021) created wireless biosensing device using CD (HA)/TiO2/Cu^2+^ coated surfaces to detect cancer cells (Entry-10 of Table 1). The biosensor uses carbonized hyaluronic acid CD (HA) as probes and is responsive to pyrophosphates (PPi) and alkaline phosphatase (ALP) concentrations. These substances are found in higher levels in cancer cell lines as compared to normal cells. These CD (HA)/TiO2/Cu^2+^ nanoassemblies has antifouling properties when exposed to visible light, which improves the accuracy of cancer cell detection by preventing unwanted biological materials from interfering. The MDCK cells were cultured on substrates coated with CD (HA)/TiO2/Cu^2+^ and exposed to visible light until the cells completely detached. Interestingly, no notable alterations were observed in the electrochemical properties and FL intensity. Conversely, when the HeLa cells were detached from the surface, there was an observed rise in resistance and FL intensity. This results shows that the CD (HA)/TiO2/Cu^2+^ exhibits great potential for investigating cell-surface interactions and cancer cell detection.

**TABLE 1:** Summary of anticancer activities of Cu, Ag and Au Carbon Dots covered in this review.

Entry No.	Description	Precursor	Application	Reference
Copper-doped-CDs				
1	Cu-CD NanoSheets	*o*-phenylenediamine and L-cysteine; Cu ions	Multimodal imaging guided Photothermal Cancer Therapy	Bao et al. (2018)
2	Cu,N-CDs nanodots	EDTA.2Na; CuCl_2_	B16 cell imaging and therapy	Guo et al. (2018)
3	CBQD-Cu NC	Spinach leaves; Copper ions	*In vivo* Biothiol imaging and Enhanced PDT of mice tumor	Liu et al. (2019)
4	Cu-CDs	Poly (acrylic acid); Cu (NO_3_)_2_	HeLa and SH-SY5Y MCs cells FL imaging and inhibition of MCs growth	Wang et al. (2019a)
5	γ-PGA@GOx@Mn, Cu-CDs Nanomaterial	Citric Acid; CuCl_2_	*In vivo* 4T1 cell imaging and therapy, *In vitro* PTT/PDT therapy of Tumor	Liu et al. (2020b)
6	CuO@CNSs-DOX nanoplatforms	Glucose; CuCl_2._ 2H_2_O	FL imaging of 4T1 cell, apoptosis via enhanced antitumor efficacy by combined therapies (PTT, CDT, CT)	Jiang et al. (2020)
7	Cu/CC NPs Nanoassembly	n/a	*In vivo*/vitro FL imaging and Synergistic Cancer therapy of A549 and 4T1 cells by (PTT, PDT, CDT)	Sun et al. (2020)
8	CuSCDB@MMT7 NC	Saccharomycetes; CuCl_2_	Cancer cell Targeting, Enhanced Antitumor. (PTT)	Yu et al. (2020)
9	CQDs/Cu_2_O Composite	Glucose; CuSO_4_	cancer ovarian (SKOV3) cell death and antiangiogenic activity	Chen et al. (2021)
10	CD (HA)/TiO2/Cu2+ NPs biosensor	Mango; CuCl_2_	HeLa cell detection	Giang et al. (2021)
11	CuO NPs-CNPs colloidal NPs	Graphite pellets; Copper suspension	Antiproliferative Actions against Breast Cancer Cell line (MCF-7)	Mohammed et al. (2022)
12	Cu-CDs	Alcea Leaf; CuSO_4_	Thermal ablation of 4T1 cancer cells	Najaflu et al. (2022)
Silver-Doped-CDs				
13	CD-Ag@ZnO NC	Acetic Acid; (AgNO_3_), (ZnNO_3_)	FL imaging and apoptosis in MCF-7 and A549 cancer cells.	Sachdev et al. (2015)
14	CyOH–AgNP/CD Nanophotosensitizer	Citric Acid; AgNO_3_	4T1 cell imaging and antitumor PDT	Liu et al. (2020c)
15	CD@AgNPs NC	Sweet lemon Peels; AgNO_3_	anticancer activity against MCF7 breast cancer cells	Ghosal et al. (2020)
16	Ag-CDs Colorimetric Sensor	Citric Acid; AgNO_3_	lactate sensing and imaging in 4T1 breast cancer cells	Park et al. (2021)
17	Ag@CDs Nanoconjugate	Citric acid; AgNO_3_	Imaging and apoptosis in HeLa cells	Priyadarshini et al. (2022)
Gold-Doped-CDs				
18	Fe3O4@PC-CDs-Au hybrid NP	Acetone; Fe(C_5_H_5_)_2_, HAuCl_4_	B16F10 cell imaging, Drug delivery and high Photothermal conversion efficiency	Wang et al. (2015)
19	C-dots–AuNPs–Cys conjugates	n/a	Detecting tumor in Hela cell using light and electricity	Liu et al. (2015)
20	AuCDs	Glucose; HAuCl_4_	FL imaging of MCF-7 and UMR-106 cells	Zhang et al. (2016)
21	GCDs NC	Citric Acid; HAuCl_4_	cytosensing of metals in cancer A549 cells	Abdelhamid et al. (2017)
22	Au/GdC NC	N-acetyl-L-cysteine; (HAuCl_4_), (GdCl_3_)	MRI contrast and PTA therapy agent	Gedda et al. (2017)
23	Au@C/CaP NPs	Polyacrylic acid; (Ca(OH)_2_), (Na_2_HPO_4_)	CT imaging contrast agent, drug delivery and Synergistic chemo-photothermal therapy	Wang et al. (2017)
24	C-dots@Au nanoflowers	Citric Acid; HAuCl_4_	HeLa cell imaging and Photothermal therapy	Hou et al. (2018)
25	(CQDs/Au) NC	Glucose; HAuCl_4_	Detection of pancreatic tumor marker (CA 19-9)	Alarfaj et al. (2018)
26	C-dots-Ab AuNPs/PAMAM/aptamer	Histidine; HAuCl_4_	Immunosensor for detection of breast tumor marker (CA 15-3)	Mohammadi et al. (2018)
27	MitoCAT-g	Citric acid; HAuCl_4_	Mitochondrial damage and apoptosis in HepG-2 cancer cell	Gong et al. (2019)
28	CD/AuNP	CDs Purchased; HAuCl_4_	Detection of MUC1 (Tumor Marker)	Wang et al. (2019b)
29	AuNP-peptide-CDs nanobiosensor	Chitosan	Detection of Matrilysin a salivary gland cancer biomarker (MMP-7)	Behi et al. (2020)
30	Au@CDs nanoalloys	Citric Acid; HAuCl_4_	Detection of MUC1-positive MCF-7 cells in serum	Liu et al. (2020a)
31	AuNP@CDs inorganic nanoflares-DNAzyme, APCD	citric acid; HAuCl_4_	Detection of exosomal miRNAs miR-133b and miR-135b	Zhang et al. (2022)
32	Au@CDs nanohybrids	Citric acid; HAuCl_4_	Tumor catalytic therapy, apoptosis in 4T1 cell	Li et al. (2022)

#### 2.1.2 Cancer detection and imaging based on Ag-CDs

**FIGURE 2:**
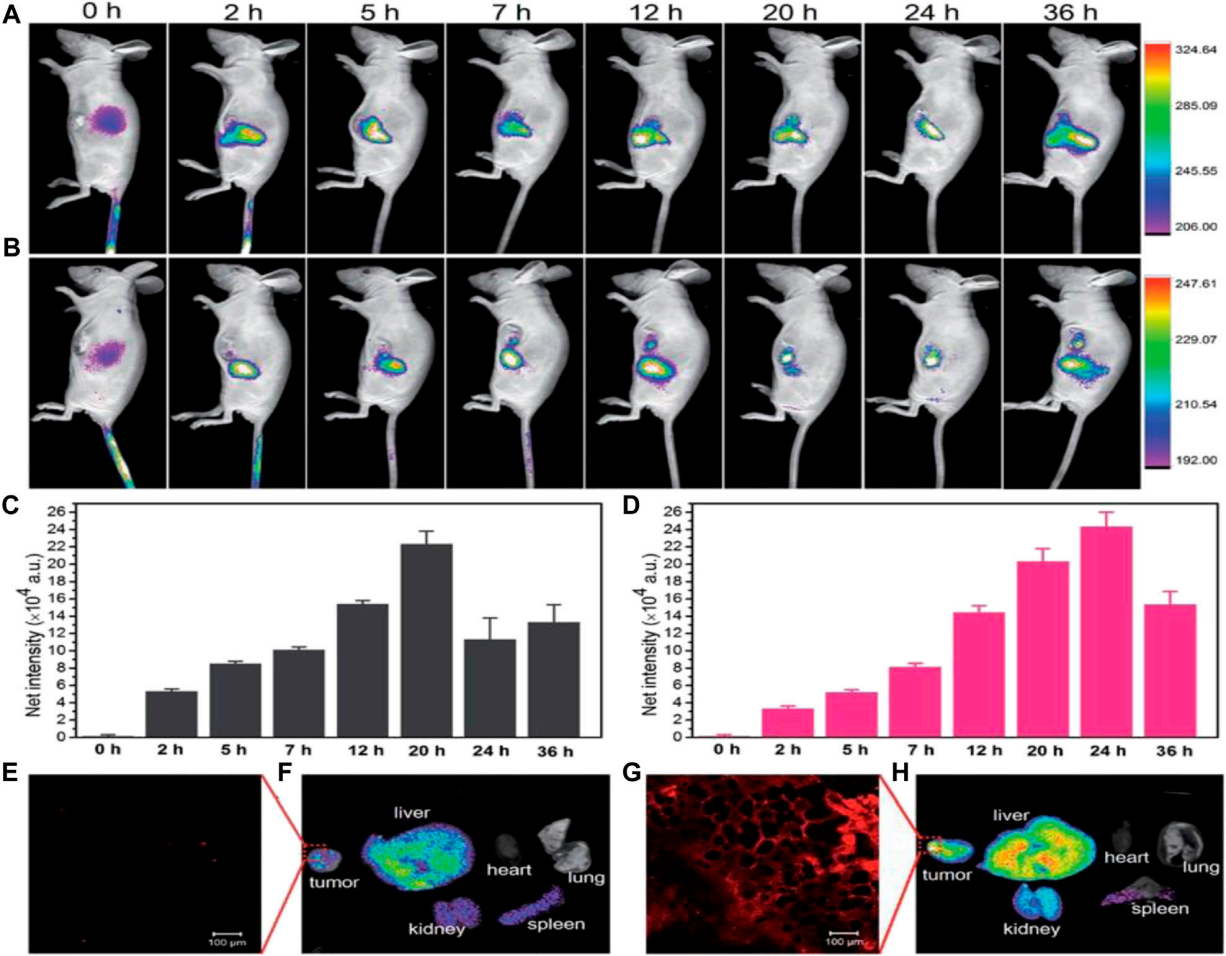
*In vivo* fluorescence images of CyOH **(A)** and CyOH–AgNP/CD **(B)** at different time points after intravenous injection. The fluorescence intensity of tumors with CyOH **(C)** and CyOH–AgNP/CD **(D)** at different time points. Confocal fluorescence microscopy image of tumors after treatment with CyOH **(E)** and CyOH–AgNP/CD **(G)**, lex ¼ 633 nm, and lem ¼ 650–750 nm. Biodistribution of CyOH **(F)** and CyOH–AgNP/CD **(H)** in tumor-bearing mice after 36 h. Reproduced from reference (Liu et al., 2020).

Sachdev et al. (2015) successfully synthesized a CD-Ag@ZnO NC that demonstrates promising potential for direct FL monitoring of cellular uptake in both A549 and MCF-7 cancerous cells (Entry-13 of Table 1). The NC allows for the concurrent green FL emission of CDs, thereby eliminating the need for fluorescent organic dyes to track the distribution of CD-Ag@ZnO NC. The technique of FL microscopy and AAS were utilized to evaluate the qualitative/quantitative aspects of cell absorption. Furthermore, they employed FL and SEM to examine distinctive nuclear and morphological alterations during apoptosis. Liu et al. (2020) have developed a highly biocompatible and low-toxicity NC, [CyOH–AgNP/CDs], which acts as a nano-photosensitizer and can be used for targeted tumor imaging (Entry-14 of Table 1). They have also conducted *in vivo* FI studies to investigate the tumor accumulation capacity of CyOH and [CyOH–AgNP/CDs]. The findings demonstrate that the near-infrared FL signals emitted by CyOH showed a progressive increase at the tumor site, peaking at 20 h after injection as shown in (Figures 2A, C). Conversely, in the case of [CyOH–AgNP/CDs], the FL signal at the tumor site reached its max intensity at 24 h following-injection (Figures 2B, D). Furthermore, confocal FL microscopy images showed (Figures 2E) weak FL for CyOH and (Figures 2G) strong FL for [CyOH–AgNP/CDs] in the tumor. This work demonstrates the potential of the [CyOH–AgNP/CDs] NC as highly effective and focused tool for tumor visualization, with low toxicity and high biocompatibility.

Priyadarshini et al. (2022) synthesized Ag@CDs nanoconjugates and investigated their potential use in biomedical applications (Entry-17 of Table 1). They found that the nanoconjugates were taken up by HeLa cells, as demonstrated by blue FL in the cells observed through confocal imaging. In addition, they measured the generation of reactive oxygen species in HeLa cancer cells treated with nanoconjugates using FI with DCFHDA dye. The internalization of CDs and Ag@CDs into cells was assessed by exposing the cells to a concentration of 50 μg/mL. Remarkably, this concentration was found to be only 1/2 of the concentrations that exhibited toxicity towards the cells. Confocal imaging data provided compelling evidence of the internalization of nanoconjugates within HeLa cells, as indicated by the presence of a distinct blue FL signal. Overall, the study demonstrated the potential of Ag@CDs nanoconjugates in various biomedical applications, including live cell imaging.

#### 2.1.3 Cancer detection and imaging based on Au-CDs

Wang et al. (2015) synthesis a type of hybrid NP, Fe3O4@PC-CDs-Au that have very potential uses in drug delivery, cell imaging, and cancer therapy (Entry-18 of Table 1). The efficacy of these NPs was tested on mouse melanoma B16F10 cells, which showed strong FL when exposed to various light wavelengths, attributed to synergistic effect of the FL CDs and Au nano-crystals in the carbon shells. Also, the cellular imaging function of the NPs was evaluated using confocal microscopy, which indicates that the NPs had the capability to penetrate the intracellular space and illuminate the cells with intense FL. The Z-scanning confocal FI of B16F10 cells following incubation with Fe_3_O_4_@PC-CDs-Au NPs provided additional evidence of the vibrant FL observed in the cytoplasm surrounding the cell nucleus upon excitation with a 488 nm laser. The images obtained from this evaluation demonstrated that Fe_3_O_4_@PC-CDs-Au NPs can surpass cell barriers and produce multicolor images of cells under laser excitation. The confocal images also showed that the NPs demonstrated Possessing exceptional photostability as an optical indicator, rendering them well-suited for extended cellular imaging purposes. Liu et al. (2015) design a CDs and cysteamine capped AuNP cytosensing platform (CDs–AuNPs–Cys) which proposes a novel method for detecting tumor cells using light and electricity without any labels or probes with high sensitivity and specificity (Entry-19 of Table 1). HeLa cancerous cells were utilized as the representative analyte model. The method uses CDs and AuNPs that can transfer energy between them when they are attached to different receptors on the cell surface and close to each other via the resonance energy transfer (RET). This energy transfer affects the electric current generated by the CDs when they are exposed to light. By measuring this current, they can tell how many cells are present on the surface of an electrode. The RET also enhances the photoelectrochemical signal of the CDs, which can be measured by a device called a photoelectrochemical Cytosensor. These advancements shows the promise of detection and diagnosis of cancer at early stages. Zhang et al. (2016) developed an Au-CDs composed of CDs and Au nanoclusters (Entry-20 of Table 1). They evaluate the cytotoxicity and cellular imaging capabilities of these Au-CDs. The results revealed that the Au-CDs exhibited biocompatibility and minimal toxicity to both cancerous and normal cells when utilized at concentrations lower than 833 μg/mL. Moreover, the Au-CDs were capable of penetrating live cells within 10 h and producing diverse colored emissions when imaged to a sole 405 nm laser stimulus. The study suggests that Au-CDs hold potential applications as multiplexed bioimaging agents for both cancer and normal cells. Specifically, under single excitation the Au-CDs were able to facilitate dual FL visualization of MCF-7 and UMR-106 cells which are breast cancerous and normal rat osteoblast cells respectively. Abdelhamid et al. (2017) fabricated modified CDs with AuNPs (Entry-21 of Table 1). The NC can be used for cytosensing of biological metals such as Fe and Cu in cancer cells (A549). The NC demonstrated exceptional ultraviolet absorption properties that aligned precisely with the N_2_ laser’s wavelength, allowing for selective detection of different metals in cancer cells with high sensitivity and accuracy. Mefenamic acid served as both a chelating agent and a co-matrix for mass spectrometry purposes, which further improving the efficiency of metal detection. Gedda et al. (2017) successfully synthesized a NC consisting of AuNPs and Gd-CDs (Entry-22 of Table 1). They further assessed the capabilities of this NC as a contrast agent for MRI. Gd containing NCs are commonly utilized as positive contrast agent in magnetic resonance imaging because they have exceptional magnetic characteristics that enable it to interact with protons in water molecules, resulting in a brighter image. They used an electron paramagnetic resonance (EPR) spectrometer to confirm the presence of Gd in the NC, and then conducted MRI experiments to measure the NCs longitudinal relaxivity (r1), which is a measure of how effectively the NC interacts with water protons to produce a contrast effect in MRI. The results showed that the Au/Gd-CDs NC had a significantly higher r1 value compared to a clinical contrast agent, Magnevist, indicating that it has strong T1 contrast ability. The elevated r1 value observed in the Au/Gd-CDs NC can be attributed to its compact size, leading to enhanced interactions between Hydrogen protons and Gd^3+^ ions. Furthermore, the NCs extensive specific surface area amplifies the dipole-dipole interactions b/w H protons and Gd^3+^ ions. Wang et al. (2017) synthesized the Au@C/CaP NPs, Consisting of an Au-core, C-shell, and Ca-Phosphate coating (Entry-23 of Table 1). The NPs were assessed as a contrast agent for computed tomography imaging, demonstrating an elevated X-ray absorption coefficient compared to conventional iodine agents, indicating a promising alternative for CT imaging. This work demonstrated a strong linear correlation (R2 value of 0.9947) between the Hounsfield units (HU) values and the Au concentration in the Au@C/CaP NPs, indicating their effectiveness as positive CT visualization contrast agents. The effectiveness of CT signals was further confirmed as their intensity increased with higher concentrations of Au. Moreover, the study revealed that the Au@C/CaP NPs did not retain any significant effect on cell viability when used alone or either with laser irradiation, as demonstrated by green FL (indicating live cells). However, when utilized alongside the exposure of an 808 nm laser beam, the Au@C/CaP NPs caused cell death in HeLa cells, as demonstrated by red FL. These findings indicate that Au@C/CaP NPs exhibit a promising and safe contrast agent for CT imaging. Which can help doctors to locate tumors and other disease. (Hou et al. (2018) have developed CDs@Au nanoflowers that exhibit dual-modal imaging capabilities for PA and FL imaging (Entry-24 of Table 1). The researchers conducted experiments on HeLa cells and found that CDs@Au nanoflowers exhibited intracellular red-emissive FL when excited by 543 nm. The results were visualized through confocal imaging and confirmed by DAPI staining technique combined with Z-stack visualization, which showed that CDs@Au nanoflowers were localized inside the cytoplasm. Additionally, they performed PA imaging experiments on an agarose gel phantom filled with CDs@Au nanoflowers at different concentrations, ranging from 50 to 300 μg/mL. The NIR region facilitated efficient absorbance of PA signals, resulting in their strong intensity. Additionally, the concentration-dependent PA curves of CDs@Au nanoflowers exhibited a linear correlation with a coefficient of determination (R2) of (0.92). These results demonstrate the potential of CDs@Au nanoflowers as a simultaneous FL and PA imaging agent for tumor diagnosis. Alarfaj et al. (2018) introduced an innovative approach for the detection of CA 19-9, a biomarker for pancreatic tumors (Entry-25 of Table 1). This method involved the utilization of a NC comprising CDs and Au. The immobilization of NCs was performed by antibody-horseradish peroxidase (Ab–HRP) formed CDs/Au–Ab–HRP. Simply it is a new way to make tiny particles that can glow when they detect a substance called CA 19-9 in blood samples. The method utilized peptide bonds to effectively capture the specific CA 19-9 antigen in human serum. This was achieved through a sandwich capping antibody-antigen-antibody reaction, enabling the immobilization of the antigen within the NC. This new technique could potentially save lives and reduce healthcare costs, by providing a faster and more cost-effective way of diagnosing pancreatic cancer. Mohammadi et al. (2018) have developed a CDs labeled with CA15-3 antibody and AuNP aptamer-based FRET immunoassay CDs-Ab–AuNPs/PAMAM/aptamer for the responsive and efficient tracing of mammary tumor biomarkers CA15-3 in various physiological samples (Entry-26 of Table 1). This method has the potential to replace or enhance existing technologies such as ELISA. When CA15-3 antigen is present in serum or cancer cell, it binds to the antibody-antigen-aptamer complex, which brings the CDs and AuNPs closer together, resulting in decreased FL intensity. The immunoassay has a high sensitivity, capable of detecting CA15-3 at extremely low concentrations of 0.9 μU/mL. Additionally, it is effective in identifying MDA-MB-231 cancerous cells within a concentration range of 1,000 to 40,000 cells, with a detection limit as low as 3 cells per 10 μL. Wang et al. (2019) presents an innovative technique to measure MUC1, a protein found on some cancer cells, using anti-MUC1 aptamers and AuNPs combined with CDs. By using inner filter effect b/w AuNPs and CDs, the method is highly selective to detect MUC1, the CDs brightness is diminished due to the filter effect in the presence of the Au particles (Entry-28 of Table 1). However, upon the addition of MUC1, the aptamers bind to it, separating them from the Au particles with restoring the CDs brightness. By quantifying the CDs brightness, the amount of MUC1 present can be calculated. This innovative method has promising potential for cancer diagnosis. Behi et al. (2020) have successfully designed nanosensing platform (Nanobiosensor) AuNPs-peptide-CDs for the rapid, sensitive and highly precise method for detecting matrilysin, a biomarker associated with salivary gland cancer (Entry-29 of Table 1). The detection platform utilizes peptides that can be digested by matrilysin, connecting AuNPs and CDs. The nanobiosensor demonstrates an impressive capability to detect minute amounts with high sensitivity, detecting particles as small as 39 nm. Additionally, it boasts a remarkably fast response time of approximately 30 s. These features make it an encouraging candidate for the noninvasive identification of tumors in their initial stages, offering great potential in the field of early tumor detection. The AuNPs-peptide-CDs complex exhibits excellent optical properties, showcasing significant FL quenching effects. When the peptide is cleaved by matrilysin, the CDs detach from the surface of the AuNPs. This leads to the rapid generation of detectable violet and visible FL signals. These results are a major step forward in developing advanced biosensors for early-stage tumor detection, with significant potential for cancer diagnosis. Liu et al. (2020a) have synthesized a new type of nanoalloy, namely Au@CDs, which exhibits exceptional electrochemiluminescence (ECL) efficiency (Entry-30 of Table 1). In order to enhance the identification of MUC1-positive MCF-7 cancerous cells, which are a type of circulating tumor cells frequently present in the bloodstream of cancer patients, they also utilized a technique involving the attachment of MUC1 aptamer, a human mucin1 protein, onto the surface of Au-coated CDs. This work investigated the ECL Cytosensor performance under optimal conditions, testing its ability to detect different levels of MCF-7 cell concentrations accurately. During their observations, it was noticed that the (ECL) signal showed a gradual decrease as the concentration of cells increased. Additionally, they observed a positive linear correlation between the ECL signal and the logarithm of MCF-7 cell concentration. Specifically, within the concentration range of 100-10,000 cells/mL. Importantly, the calculated limit of detection (LOD) for MCF-7 cell concentration was found to be 34 cells/mL, which are lower than many previous studies. These findings demonstrate the remarkable sensitivity of the Cytosensor in detecting MCF-7 cells at an extremely low concentration level. Potentially enabling earlier detection and treatment of cancer patients. Notably, this approach is comparatively straightforward and does not require separation or labeling procedures, making it an attractive option for CTCs detection. Zhang et al. (2022) developed a cutting-edge technique for detecting bladder cancer-related exosomal miRNAs simultaneously (Entry-31 of Table 1). Their method combines inorganic nanoflares with DNAzyme walkers, enabling the precise and accurate detection of miRNAs in urine samples with high sensitivity and specificity from bladder cancer patients. Specifically, the inorganic nanoflares consist of AuNPs modified with CDs labeled substrates and DNAzyme strands (AuNPs@CDs inorganic nanoflares-DNAzyme APCD). By integrating these components into a single assay, they successfully attained an impressive sensitivity level for detecting a solitary miRNA, reaching the femtomolar range. Moreover, they established a broad linear detection range spanning from 50 fM to 10 nM. This innovative technique has the potential to revolutionize the early detection of bladder cancer.

### 2.2 Phototherapy

Phototherapy whether PDT or PTT, is a type of noninvasive therapy (not invading adjacent healthy cells, tissues and blood vessels) which changes the irradiating light into a variety of ROS (e.g., O_2_ •−, •OH etc.), heat with the aid of photosensitizers and induces local apoptosis of cancer cell lines. CDs have attained enormous attraction as brilliant phototherapeutic agents because of their exclusive optical characteristics, enhanced photostability and high water-solubility. The therapeutic effects of CDs can be dangerously blocked in oxygen-dependent PDT due to rapid oxygen consumption and hypoxic (low oxygen) tumor microenvironment. This may result in inevitable drug resistance or tumor metastasis. To tackle this problem NCs for light-driven water splitting have been developed to improve the intra-tumoral oxygen level and finally reverse the hypoxia-triggered PDT resistance and tumor metastasis (Zheng et al., 2016). Apart from anticancer phototherapies, drug delivery efficiency with advantage of therapy can be achieved by combining imaging tools with drugs or genes to in the form of imaging-guided nanohybrids (Liu et al., 2020). Carrying medicine to a specified location and then its release in uninterrupted fashion is the key to efficient safe treatment. Therefore, this is a crucial step to enhance the localized therapy with minimum side effects to healthy and non-cancerous cells. CDs with aid of their excellent fluorescent properties during efficient therapy can rapidly visualize drug accumulation and activities in the cancer cell lines (Gao et al., 2020). CDs have exhibited potential clinical imaging and drug delivery applications during brain cancer and CNS diseases (Shao et al., 2017).

In the following section, Cu-, Ag-, and Au-doped CDs are summarized which have been reported to apply during drug delivery and cancer therapy treatments.

#### 2.2.1 Cancer therapy based on Cu-CDs

Bao et al. (2018) have developed Cu/CDs-crosslinked NSs (Entry-1 of Table 1). These NSs demonstrate exceptional optical absorption in the NIR range and an impressive photothermal conversion efficiency of 41.3% at 808 nm after modification with PEG. This high conversion efficiency enables it to convert NIR light energy to hyperthermia rapidly and efficiently, which can be used to kill cancerous cells. In this study, the cell toxicity of the CuCD NSs was evaluated using various cells, including MCF-7, HepG2, AT II, A549 and L02 cells. The results showed that the cellular survival rates exceed 80% even when exposed to Cu concentration of 30 μg/mL, indicating low toxicity. Additionally, no hemolysis was observed when RBCs were treated with various concentrations of Cu^2+^. Therefore, *in vivo* and *in vitro* the PEG-modified CuCD NSs are suitable for PTT. The NSs were also found to enhanced therapeutic effectiveness through laser-induced delivery into the cytosol, escape from the lysosomes, and targeting of the nucleus like properties. Guo et al. (2018) investigated the potential use of Cu,N-CDs for cancer treatment, examining their effectiveness in both *in vivo* and vitro (Entry-2 of Table 1). The study focused on evaluating the ability of these CDs to serve as a therapeutic agent against cancer, using melanoma B16 cells. The Cu,N-CDs possess NIR absorption properties that generate heat and ROS necessary for PTT and PDT. The production of ROS, particularly ^1^O_2_, is crucial for PDT promoting cancer cell apoptosis. To determine the ROS production capacity of Cu,N-CDs, the chemical trapping agent 1,3-diphenylbenzofuran (DPBF) was used, and the results indicated, Cu,N-CDs generate ROS efficiently when exposed to laser irradiation of 800 nm. The phototherapeutic effect of Cu,N-CDs was evaluated *in vitro* by incubating B16 cells with varying concentrations of Cu,N-CDs and subjecting them to laser exposure of 800 nm for 10 min. The results demonstrated a concentration-dependent reduction in cell viability, from 88% to 20%, proposing toxicity arising from both the thermally-induced photothermal effects and the generation of ROS triggering photodynamic effects. Overall, the study suggests that Cu,N-CDs can inhibit melanoma tumors in mice through synergistic PT and PDT, providing a promising therapeutic approach for cancer therapy. Liu et al. (2019) prepared “chlorophyll-inrich biomass QDs” and formed CBQD-Cu NCs by adding Cu (Entry-3 of Table 1). The CBQD-Cu have emerged as an exceptionally potent form of nanomedicine utilized for the identification and therapy of tumors. *In vivo* study demonstrated that these CBQD-Cu NCs exhibit dual enhanced PDT of tumors. When tested on mice with tumors, the NCs successfully treated the tumors, with no detectable infected tissues found after 12 days of complete treatment, as observed through optical microscopy. The attachment of chlorophyll and Cu^2+^ on the surface of the CBQDs also played a crucial role in enhancing PDT. This binding reduced difference in energy level of the chlorophyll molecules, which led to an increase of ROS under NIR irradiation. This increase in ROS production ultimately resulted in enhanced PDT, making these CBQD-Cu NCs a promising new nanomedicine for PDT of tumors. Wang et al. (2019) reports that the Cu-CDs synthesized in this study exhibited a higher quantum yield 36% of ^1^O_2_ and inhibition the growth of 3D multicellular spheroid (Entry-4 of Table 1). Indicating their potential to promise visualizing-guided PDT agent. They used EPR spectroscopy to validate the effectiveness of PDT by detecting the presence of singlet oxygen (^1^O_2_). The EPR analysis revealed an enhancement in the signal of Cu-CDs after 12 min of exposure to light, indicating successful PDT activation. Conversely, the signals of CDs and water were relatively diminished under both dark and light conditions. Therefore, the results from the EPR spectroscopy demonstrated an amplified signal for Cu-CDs while observing weakened signals for CDs and water in the presence of light during PDT. The Cu-CDs showed good photoinduced cytotoxicity and can be used as a versatile photodiagnostic and therapeutic tool in various biological applications. Liu et al. (2020) have synthesized a novel, biodegradable NP composite that can combine four different approaches to cancer treatment like starving therapy, PDT, PTT, and immune based therapy (Entry-5 of Table 1). The NPs, known as γ-PGA@GOx@Mn, Cu-CDs NPs, have an exceptional capacity to selectively target malignant cells and remain within the acidic microenvironment of tumors for a prolonged time. When these NPs exposed to 730 nm laser, they exhibit both photothermal and photodynamic effects, with the added advantage of generating hydrogen peroxide (H_2_O_2_) *in situ* to reduce tumor hypoxia and improve *in vivo* PDT. Thus, by combination of these therapies results in excellent tumor inhibition, as demonstrated by the high collection of NPs within the tumor tissues, coupled with synergistic effect of starving therapy, PDT and PTT. Moreover, when combined with anti-PDL1 checkpoint blockade treatment, this NP-based combination therapy is able to directly remove primary tumors and target metastatic tumors, leading to the suppression of distant tumors. Jiang et al. (2020) have developed a versatile Nano platform called CuO@CNSs-DOX, which can be used for the combining therapies of PTT, CDT, and CT (Entry-6 of Table 1). During synthesis CuO are adsorbing on carbon nanostructures surface, an enhancement was observed in the efficiency of photothermal conversion for NPs, resulting in an increase from 6.7% to 10.14%. This enhancement can be attributed to the electron transition that occurs between C-2p and Cu-3d. CuO also used as a CDT agent, which is capable of selectively releasing Cu^2+^ ions at the tumor site. These ions can trigger the generation of Hydroxyl radicals (•OH) through Haber-Weiss and Fenton-like reactions, leading to the induction of apoptosis in cancer cells. Additionally, the therapeutic drug DOX was loaded onto CuO@CNSs through electrostatic adhesion. This formulation enables the rapid release of DOX specifically at the tumor site, effectively targeting and eliminating cancer cells. The CuO@CNSs-DOX platform shows promise in improving the effectiveness of cancer treatment by utilizing the synergistic effects of PTT, CDT, and CT. Sun et al. (2020) evaluates the capability of Cu/CC NPs for cancer treatment (Entry-7 of Table 1). The study investigates the synergistic therapeutic effect of combining PDT, PTT and CDT using Cu/CC NPs. The Cu/CC NPs *in vivo*/vitro studies show excellent tumor homing capacity, high photothermal conversion efficiency, enhanced accumulation capability, and PTT efficiency. *In vitro* the cell-toxicity of Cu/CC NPs on normal and cancer cells were investigated. The cell viability was assessed utilizing a cell counting kit-8 (CCK8) assay. The findings revealed that the viability of MRC-5 normal cells remained above eighty percent even when exposed to the highest concentration of Cu/CC NPs (200 mg/mL). In contrast, the viability of 4T1 and A549 cancerous cell line decreased rapidly with increasing concentrations of Cu/CC NPs. Specifically, particularly at a concentration of 200 mg/mL, the viability of A549 cells was observed to be only 51%, while the viability of 4T1 cells was even lower, with only 38% remaining alive, indicating that the nano-assemblies had enhanced cytotoxicity towards cancer cells in comparison to normal cells. The combination therapy leads to significant tumor inhibition, with the highest efficacy observed in the trimodal CDT, PTT and PDT. Yu et al. (2020) design a hollow-structured CuS NPs that were combine with CDs and loaded with the drug bortezomib to target tumors (Entry-8 of Table 1). To increase specificity for cancer cells, the nano-composite were coated with a macrophage membrane hybridized with T7 peptide, which helped them evade the immune system and enter cancer cells through transferrin receptor-mediated endocytosis. The biocompatible and less toxic CuSCD NPs had excellent photothermal conversion efficiency when exposed to laser irradiation of 808 nm. After coating with the macrophage membrane hybridized with T7 peptide, the resulting CuSCDB@MMT7 NC showed increased specificity for cancer cells and improved immune evasion. They used CuSCDB@MMT7-triggered PTT to treat tumors, they observed an increase in tumor cell apoptosis and a decrease in metastasis. This was due to the increased heat-stability of various substrates involved in cell proliferation and survival, which are part of the ubiquitin-dependent proteasomal degradation pathway. Chen et al. (2021) developed a composite of CD and Cu_2_O and investigated its anticancer and antiangiogenic properties in various cancerous and normal cells (Entry-9 of Table 1). This finding demonstrated that CDs/Cu_2_O displayed enhanced sensitivity towards SKOV3 cells in comparison to HeLa, A549, HT-29, HCT116 cancer cells, as well as normal cells. The IC50 value recorded for SKOV3 cells was 0.85 μg mL−1, which was roughly three times lower than the IC50 values observed for the other tested cancer cells, and approximately 12 times lower than that for normal cells. Furthermore, CDs/Cu_2_O demonstrated stronger antitumor activity Compared to commonly used anticancer drugs like artesunate (ART) and oxaliplatin (OXA), the IC50 value of the CDs/Cu_2_O NCs was significantly lower. Specifically, the IC50 value for the NCs was approximately 114 times lower than that of ART and approximately 75 times lower than that of OXA. Furthermore, the CDs/Cu_2_O NCs demonstrated enhanced antiangiogenic properties in comparison to the commercial antiangiogenic inhibitor, SU5416. This was attributed to the downregulation of VEGFR2 expression. Moreover, the CDs/Cu_2_O NCs exhibited the ability to modulate angiogenesis-related genes in SKOV3 cells, specifically impacting genes such as Maspin and TSP1, thereby effectively suppressing angiogenesis Mohammed et al. (2022) synthesize CuO NPs-CNPs NC by pulsed laser ablation in liquid (PLAL) (Entry-11 of Table 1). This NC consisted of CuO NPs decorated C-NPs. The potential anticancer properties of CNP-CuO NPs were investigated in a study conducted on the MCF-7 breast cancer cell line. Additionally, the biocompatibility of these NPs was assessed to ensure their safe application. The results demonstrated that CNP-CuO NPs exhibited a significantly higher cytotoxicity against MCF-7 cells compared to CNPs alone, with the highest anticancer effects reaching almost 85%. These findings suggest that CNP-CuO NPs have high potential as an anticancer agent. Najaflu et al. (2022) have successfully synthesized 3 nm Cu-CDs with a green synthesis approach (Entry-12 of Table 1). The resulting Cu-CDs nanospheres demonstrated strong thermal ablation effects on 4T1 cells upon exposure to an 808 nm NIR laser irradiation. Importantly, the Cu-CDs showed low cytotoxicity *in vitro*, and their photothermal conversion efficiency was measured to be 39.3%. These findings suggest that Cu-CDs can be internalized by cells causing induce cell thermal death when exposed to 800 nm NIR laser irradiation, making them a promising candidate for cell PTT.

#### 2.2.2 Cancer therapy based on Ag-CDs

Sachdev et al. (2015) developed a CD-Ag@ZnO NCs, which have significant potential in effectively monitoring the internalization of substances by MCF-7 and A549 cancer cells, as well as triggering programmed cell death (apoptosis) in these cells (Entry-13 of Table 1). *In vitro* studies have shown that the concentration-dependent cytotoxic effects of CD-Ag@ZnO NCs are attributed to the induction of apoptosis, which is accompanied by a notable rise in the generation of intracellular ROS. The elevation in ROS levels is closely linked to mitochondrial dysfunction, which subsequently triggers the initiation of apoptosis. These findings suggest that CD-Ag@ZnO NCs could be a potential candidate for cancer treatment. Liu et al. (2020) developed a biocompatible and low-toxicity NCs, [CyOH–Ag-NPs/CDs], Act as a nano-photosensitizer with high efficiency for PDT (Entry-14 of Table 1). They conducted experiments on mice in groups with 4T1 tumors to evaluate the therapeutic efficacy of this NC. The growth of tumors was partially suppressed in both the AgNP/CDs and CyOH groups. However, the CyOH–Ag-NPs/CDs group demonstrated remarkable tumor reduction, which can be attributed to the enhanced production of singlet oxygen (^1^O_2_) by this specific NC. The CyOH–Ag-NPs/CDs nano-photosensitizer showcased numerous benefits, such as a substantial production of singlet oxygen, targeted accumulation in mitochondria, improved penetration into tissues under 660 nm laser irradiation, and enhanced specificity towards tumor cells. In comparison to the CyOH dye or AgNP/CDs nanohybrid, it displayed a more potent antitumor effect. Ghosal et al. (2020) have successfully synthesized a natural polysaccharide derived CDs based AgNP NC and exhibits its anticancer activity against breast cancer MCF-7 cells (Entry-15 of Table 1). The synthesis method was *in situ*, facile and green, making it an eco-friendly approach. The anticancer effect of the CD@AgNPs was found to be dose-dependent and attributed to the generation of intracellular ROS leads to cell apoptosis. In addition to its anticancer properties, CDs demonstrated exceptional optical properties, characterized by excitation-dependent multicolor FL emission and remarkable photostability. Priyadarshini et al. (2022) synthesized a nanoconjugate called Ag@CDs, made up of Ag and CDs, and tested its efficacy against HeLa cells, a type of cervical cancer cell (Entry-17 of Table 1). A study revealed that the utilization of Ag@CDs resulted in the suppression of HeLa cell proliferation, exhibiting an IC50 value of approximately 50 ± 1:0 μg/mL, while also inducing apoptosis. The observed mechanism behind this effect can be attributed to the production of ROS, which can induce harm to cellular structures and activate pathways leading to cell death. This indicates that Ag@CDs has a potential to be a promising anticancer drug with potent therapeutic effects, making it a promising candidate for further development and testing.

#### 2.2.3 Cancer therapy based on Au-CDs

Wang et al. (2015) developed a Fe_3_O_4_@PC-CDs-Au NPs offer therapeutic potential and drug delivery carriers because of their remarkable ability to convert light into heat at a highly efficient rate and drug loading capacity (Entry-18 of Table 1). To test the photothermal performance of Fe_3_O_4_@PC-CDs-Au NPs they studied PT effect of water, aqueous dispersion of Fe_3_O_4_@PC-CDs template NPs and Fe_3_O_4_@PC-CDs-Au hybrid NPs under NIR irradiation, and the results show that upon exposure to same NIR irradiation for 5 minutes, the temperature of water increased by 5°C, followed by Fe_3_O_4_@PC-CDs 25°C and Fe_3_O_4_@PC-CDs-Au hybrid NPs 34°C. The template NPs of Fe_3_O_4_ coated with CDs already demonstrate excellent photothermal conversion capability. Moreover, by incorporating Au nanocrystals onto the carbon shell, the photothermal effect of the resulting hybrid NPs (Fe_3_O_4_@PC-CDs-Au) can be greatly intensified. That’s why the Fe_3_O_4_@PC-CDs-Au hybrid NPs is ideal candidate for PTT. Furthermore, the NPs can be easily dispersed in water and carry drug molecules via their hydrophilic hydroxyl/carboxyl surface functional groups and porous carbon structure. They also found the loading capacity of DOX molecules into the NPs, which is approximately 71.9 wt%, which can be attributed to the various interactions between the DOX molecules and the NPs. Including π-stacking, hydrogen bonding, and electrostatic attractions. Additionally, the work mention that the drug molecule can be released from NPs can exhibit altered behavior when exposed to a magnetic field or NIR light. These NPs can be modified with targeting ligands to improve their specificity towards cancer cells. Gedda et al. (2017) synthesized a (10^-9^) composite consisting of Au and Gd-CDs (Entry-22 of Table 1). These NPs exhibit low levels of toxicity and favorable biocompatibility, even at high concentrations, as observed in both *in vitro* experiments using HeLa cells and *in vivo* experiments conducted on zebrafish embryos. Furthermore, the explored the potential application of the NC as a photothermal agent (PTA) for cancer therapy. This is attributed to the NCs capability to absorb NIR light and convert it into thermal energy. The NC was found to have a high photothermal conversion efficiency and was photostable under laser irradiation. *In vitro* experiments showed that the NC had low toxicity and could kill cancer cells in a concentration-dependent manner when exposed to NIR laser irradiation. The trypan blue staining and MTT assay results were consistent and indicated that the NC could induce cell death through localized hyperthermia. In addition, when the concentration reached 2 mg/mL or higher, the solutions exhibited the ability to raise temperatures above 42°C. This temperature elevation proved effective in eliminating tumor cells. Wang et al. (2017) synthesized Au@C/CaP NPs was produced, which are core-shell NP consists of an Au core, a carbon shell and a Ca-phosphate coating (Entry-23 of Table 1). The Au@C/CaP NPs exhibit pH- and NIR-responsive drug release properties (Mean it can carry a drug inside them and release it when they are exposed to acidic conditions (like in cancer cells) or NIR light). The Au@C/CaP NPs can also enhance photothermal conversion efficiency by heat up when absorb near infrared light, which can kill cancer cells. The study demonstrates that Au@C/CaP NPs can be loaded with DOX, an extensively employed anti-cancer medication, and can effectively deliver DOX to cancer cells under acidic or NIR stimuli. The paper also shows that the Au@C/CaP NPs can induce synergistic CT and PTT of cancer cells by combining DOX-mediated cytotoxicity and NIR-induced hyperthermia. Hou et al. (2018) developed CDs@Au that exhibit efficient PT properties under laser irradiation of 750 nm, this study also evaluates the potential of using localized hyperthermia to facilitate PTT in HeLa cells (Entry-24 of Table 1). They achieved a photothermal conversion efficiency of around 22.5% and examined the effectiveness of CDs@Au nanoflowers in inducing PTT and assessing their impact on HeLa cells. Notably, the nanoflowers exhibited minimal toxicity to HeLa cells across a range of concentrations (0-300 μg/mL), indicating their low cytotoxicity.

**FIGURE 3:**
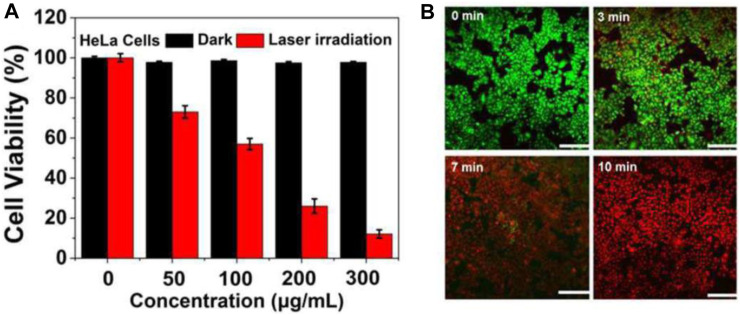
MTT assay and PTT effects reproduced from reference (Hou et al., 2018). **(A)** Relative viability of HeLa cells incubated with gradually increasing concentrations of CDs@Au nanoflowers before and after irradiation by a 750 nm laser (2 W/cm^2^) for 10 min. **(B)** Under a 750 nm laser irradiation (2 W/cm^2^) from 0–10 min, the time-dependent confocal images of HeLa cells incubated with 300 μg/mL of C-dots@Au nanoflowers and co-stained by calcein-AM/PI. Scale bar: 100 nm.

However, when HeLa cells were exposed to a 750 nm laser at a power density of 2 W/cm^2^ for a duration of 10 min, the viability of the cells decreased significantly. The decrease in cell viability was observed with increasing concentrations of CDs@Au nanoflowers, and the highest concentration tested, 300 μg/mL, resulted in approximately 90% cell death (Figures 3A). To assess the PTT efficacy of CDs@Au nanoflowers, a staining technique involving calcein-AM (green) and PI (red) was employed. The results indicated (Figures 3B) that cell death was dependent on the duration of laser irradiation. Following a 3-min irradiation, cells exhibited signs of heating caused by the CDs@Au nanoflowers. Prolonging the irradiation time to 7 min led to the destruction of the majority of cells. After 10 min of irradiation, nearly all cells were eradicated. These findings demonstrate the effective performance of CDs@Au nanoflowers in PTT, highlighting their potential application in cancer treatment.

Gong et al. (2019) developed a mitochondrial oxidative stress amplifier (MitoCAT-g), a therapeutic agent (Entry-27 of Table 1). Which has the ability to selectively target and enter mitochondria, the powerhouses of cells. Once inside, it catalyzes reactions involving oxygen and glutathione, resulting in the generation of ROS that can cause irreversible mitochondrial damage and programmed cell death (apoptosis) in cancer cells. Interestingly, *in vivo* MitoCAT-g demonstrated remarkable efficacy in inhibiting tumor growth in both subcutaneous and orthotopic HCC PDX (hepatocellular carcinoma patient-derived xenograft) models, without any toxic activity. Li et al. (2022) have developed a novel nanohybrid called Au@CDs, which consists of Au and CDs (Entry-32 of Table 1). These nanohybrids demonstrate enzyme-like catalytic activity when exposed to NIR light and have excellent SERS properties. The Au@CDs nanohybrids possess NIR-photoinduced peroxidase-like catalytic processes via a SERS strategy, which can be utilized for cancer treatments. They also have glutathione oxidase-like activities that can enhanced the effects of ROS and other cancer treatments, making cancer cells more susceptible to damage. The work also shows the nanohybrids potential for PDT by promoting apoptosis in cancerous cells in just 3 min. SERS is utilized to watch the ROS activity of the TME, and this work shows that after the NIR light source is removed for 33 min, the presence of glutathione counteracts and eliminates the ROS activity of the TME. This research has significant implications for the development of artificial enzymes to be used in therapeutic strategies targeting ROS. Additionally, it introduces a novel spectroscopic tool that can be employed to evaluate the effectiveness of catalytic therapy in treating tumors.

## 3 Conclusion

Since their discovery with less than 20 years ago, the fluorescent Carbon Quantum Dots have emerged as effective alternate to other conventional quantum dots in the biomedical applications including imaging and therapy mainly because of their easy and simple synthesis, non-toxicity, enhanced biocompatibility and outstanding optical properties including high photostability, multi-color emission based on excitation, Near Infra-Red light absorbing ability and excellent up-conversion photoluminescence. However, the interesting feature is their easy integration/doping with other metals and nanomaterials which enhances their physicochemical properties.

In this manuscript we have presented a summary of current applications of group-11 (Cu-, Ag- and Au)-Carbon Dots as innovative tools for cancer treatment. The metal-CDs nanocomposites, nanohybrids or heterostructures have shown remarkable and encouraging applications in the field of cancer theranostics. A detailed overview of the literature is given of group-11 metals-doped carbon dots having application in cancer imaging and therapy with potential candidates for clinical use. Both *in-vivo* and *in-vitro* anti-cancer studies show promising and encouraging results based on their biocompatibility, cytotoxicity and photostability. Different precursors as sources of CDs can be employed in doping, nano-composites formation, nano-hybrids formation or heterostructures with the mentioned metals.

In summary, this review demonstrates Ag-, Cu- and Au-doped-Carbon Dots as a new emerging class of C-based nano fluorescent materials for cancer diagnosis and therapy. However, from a perspective to be well-established in this direction few challenges need to be addressed.

## 4 Current challenges and future perspective

Despite their improved performance for cancer treatment based on novel methods of synthesis these group-11 metals doped carbon dots still have some challenges as follows:

1. Rapid microwave-assisted and sonochemical methods of synthesis needs to be established.
2. Although FL imaging is now well established, however, MRI, PAT and NIR based *in-vivo* and *ex-vivo* models have yet to be studied in more details.
3. Compared to photothermal therapy, photodynamic therapy has not been explored to the required extent.
4. Detailed characterization of formation mechanism of these metal-based-doped CDs using *in situ* techniques is necessary better understanding and useful application in cancer treatment.
5. To explain structure-performance correlation, more advanced techniques like SXR (synchronous X-ray radiation), TR-EPR (time-resolved electron paramagnetic resonance), SAC-STEM (spherical-aberration correction scanning/transmission electron microscopy), MALDI-TOF/MS matrix-assisted laser desorption ionization time-of-flight mass spectroscopy) needs to be used on regular basis for better understanding.
6. Regarding the excellent biocompatibility and almost zero toxicity carbon dots emitting deep red to NIR (650–1700 nm) that are excited by deep red to NIR light are desirable in future photo-theranostics in clinical applications. Therefore, more systematic research work is required in this direction.
7. There are many unanswered questions regarding different aspects of metal-doped carbon-dots that will for sure inspire multi-disciplinary research work looking into the rich future of these fluorescent nanomaterials.

## Abbreviations

CDs, Carbon Dots; NPs, Nanoparticles; NSs, Nanosheets, Nanospheres; NCs, Nanocomposites; FL, Fluorescence; FI, Fluorescence Imaging; PA, Photoacoustic; PT, Phototherapy, Photothermal; CT, Chemotherapy; PTT, Photothermal Therapy; PDT, Photodynamic Therapy; CDT, Chemo dynamic Therapy; PTA, Photothermal ablation, Photothermal agent; AAS, Atomic absorption spectroscopy; NIR, Near infrared; GSH, glutathione; CBQD, Chlorophyll rich biomass quantum dots; PGA, poly glutamic acid; Cys, Cysteamine, Cysteine; LOD, Limit of Detection; ECL, Electrochemiluminescence; PEG, polyethylene glycol; MCs, multicellular spheroids; ROS, Reactive Oxygen Specie; EDTA, Ethylene diamine tetraacetic acid; MTT, methyl thiazolyltetrazolium.
